# A concise enantioselective synthesis of the guaiane sesquiterpene (−)-oxyphyllol

**DOI:** 10.3762/bjoc.9.239

**Published:** 2013-10-08

**Authors:** Martin Zahel, Peter Metz

**Affiliations:** 1 Fachrichtung Chemie und Lebensmittelchemie, Organische Chemie I, Technische Universität Dresden, Bergstrasse 66, 01069 Dresden, Germany

**Keywords:** asymmetric synthesis, hydration, natural products, terpenes, transannular epoxide opening

## Abstract

(−)-Oxyphyllol was prepared in only 4 steps from an epoxy enone that already served as an intermediate for the total synthesis of the anticancer guaiane (−)-englerin A. A regio- and diastereoselective Co(II)-catalyzed hydration of the olefin and a transannular epoxide opening were used as the key reactions.

## Introduction

**Figure 1:**
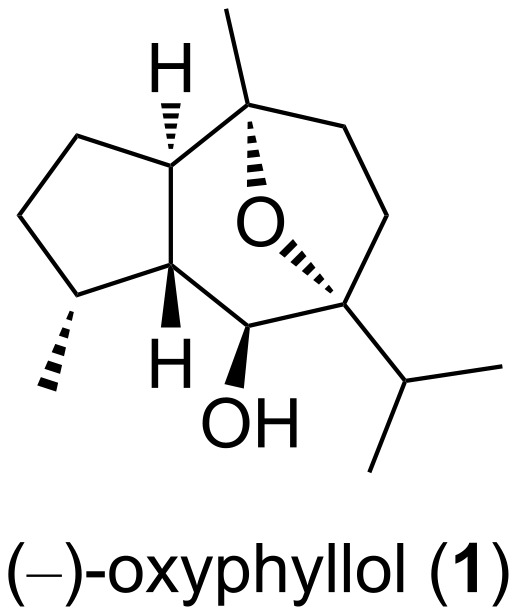
Structure of the guaiane (−)-oxyphyllol (**1**).

The hydroazulene framework is present in many natural products that are often associated with interesting biological properties [1–2]. The guaiane sesquiterpene (−)-oxyphyllol (**1**) has been isolated from the roots of the Thai medicinal plant *Phyllanthus oxyphyllus* [3]. A recent enantioselective synthesis of the unnatural enantiomer of **1** enabled a structural revision of this compound and established its relative and absolute configuration as depicted in Figure 1 [4]. During the total synthesis of (−)-9-deoxyenglerin A, compound **1** had already been prepared enantiomerically pure in 14 steps starting from (−)-isopulegol [5]. Remarkably, its cinnamate [(−)-9-deoxyenglerin A] displayed a cytotoxic activity in the μM range against several cancer cell lines [5]. Herein we report a concise enantioselective access to (−)-oxyphyllol (**1**) in a few preparatively simple operations.

## Results and Discussion

**Scheme 1:**
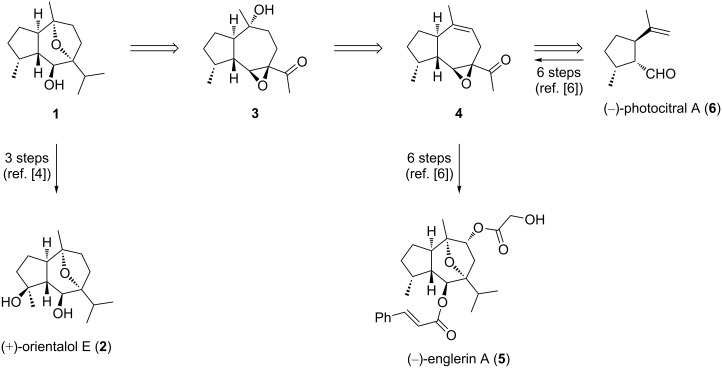
Retrosynthetic analysis for (−)-oxyphyllol (**1**) and structures of the guaiane sesquiterpenes (+)-orientalol E (**2**) and (−)-englerin A (**5**).

Recently, we developed an efficient access to the anticancer guaiane (−)-englerin A (**5**) starting from (−)-photocitral A (**6**), which in turn can be prepared in only 2 steps from commercially available (−)-isopulegol through dual catalysis [6]. Due to the structural similarity of the hydroazulene core in (−)-oxyphyllol (**1**) and **5**, we decided to use a modification of our route to **5** for the synthesis of **1** that would also constitute a formal synthesis of the related guaiane (+)-orientalol E (**2**) [4,7]. As illustrated in Scheme 1, we selected tertiary alcohol **3** as a retrosynthetic precursor for **1**. The acetyl group of **3** can be utilized to generate the isopropyl group, and a regioselective transannular epoxide opening would construct the oxygen-bridged bicyclic hydroazulene framework. Alcohol **3** was traced back to the known epoxy enone **4** that already served as an intermediate for the total synthesis of **5** [6].

**Scheme 2:**
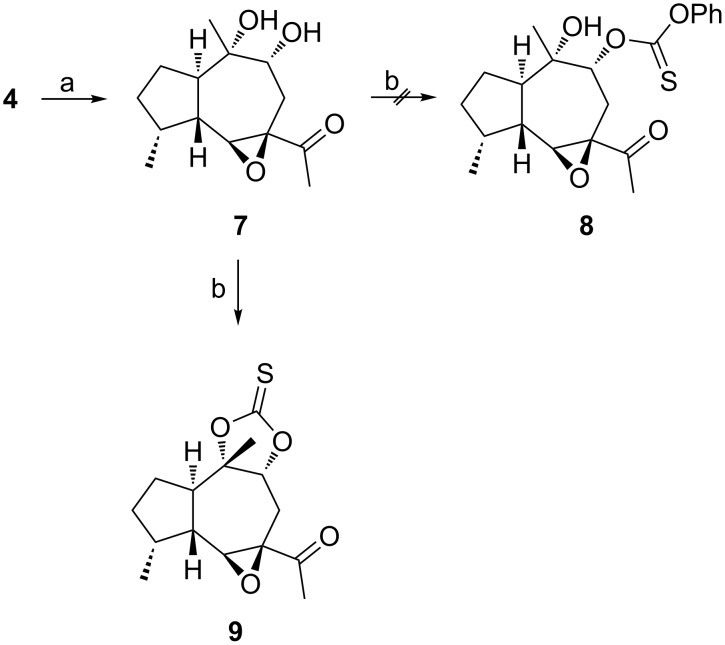
Attempted selective deoxygenation of diol **7**. a) 1 mol % K_2_OsO_4_, NMO, acetone, water, THF, rt, 97%, diastereomeric ratio = 2:1 (ref. [6]); b) PhOC(S)Cl, pyridine, CH_2_Cl_2_, 0 °C to rt, 42% **9**. NMO = *N*-methylmorpholine *N*-oxide.

As shown in Scheme 2, we first planned to use the known diol **7**, which is available by diastereoselective dihydroxylation of **4** [6]. Since a chemoselective deoxygenation of the secondary alcohol of **7** would give rise to the desired intermediate **3**, we investigated a radical defunctionalization strategy [8]. To this end, we tried to prepare the thionocarbonate **8** from **7** with phenyl chlorothionoformate [9–10]. However, all attempts at selective activation of the secondary hydroxy group led to the cyclic product **9** instead. A similar problem was already encountered earlier [11].

**Scheme 3:**
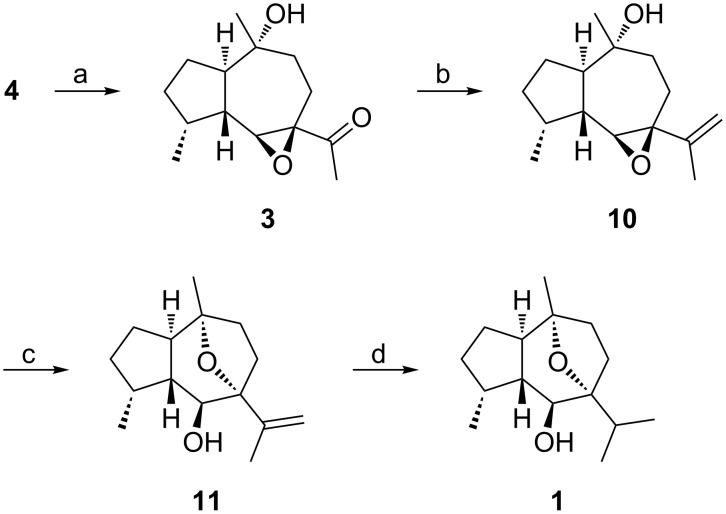
Conversion of **4** to **1**. a) 20 mol % Co(acac)_2_, PhSiH_3_, 1 atm O_2_, THF, 0°C, 82%, diastereomeric ratio = 2.4:1; b) Ph_3_P=CH_2_, THF, rt; c) 2 mol % Yb(OTf)_3_, CH_2_Cl_2_, rt, 80% (2 steps); d) 1 atm H_2_, 10% Pd/C, MeOH, rt, 98%. acac = acetylacetonate, Tf = trifluoromethanesulfonyl.

Scheme 3 depicts the efficacious completion of the synthesis of (−)-oxyphyllol (**1**) from **4**. To our delight, a direct regio- and diastereoselective Co(II)-catalyzed hydration [12] of the olefin in **4** succeeded to give the required α-stereoisomer **3** in 58% isolated yield after chromatographic separation of the minor β-alcohol. Compared to the envisaged deoxygenation route (Scheme 2), this key transformation saved 2 steps and paved the way for a final reaction sequence that was based on our synthesis of (−)-englerin A (**5**) [6]. Thus, Wittig olefination of the acetyl group in **3** afforded the sensitive vinyl epoxide **10** along with some cyclized product **11**. In contrast to the smooth transannular epoxide opening [13–14] encountered during the synthesis of **5**, an attempted complete cyclization of **10** to give **11** during acidic work-up of the methylenation reaction led to considerable decomposition. Fortunately, catalytic amounts of ytterbium triflate accomplished a high yielding formation of the oxygen-bridged bicyclic hydroazulene **11**. Finally, hydrogenation of **11** proceeded uneventfully to deliver the target molecule **1** nearly quantitatively.

## Conclusion

In summary, we have accomplished a short enantioselective total synthesis of (−)-oxyphyllol (**1**) and thus, a formal synthesis of (+)-orientalol E (**2**) as well by a modification of our previously developed route for setting up the oxygen-bridged framework present in (−)-englerin A (**5**). The reaction sequence presented herein allows the preparation of the guaiane **1** in only 10 steps with an overall yield of 22% starting from (−)-photocitral A (**6**).

## Experimental

**General information:** Tetrahydrofuran and dichloromethane were dried and purified by passage through a MB-SPS-800 device using molecular sieves. All commercially available reagents were used as received. Reactions were performed under argon atmosphere. Thin layer chromatography (TLC) was performed on Merck silica gel 60 F_254_ 0.2 mm precoated plates. Product spots were visualized by UV light at 254 nm and subsequently developed using anisaldehyde solution as appropriate. Flash column chromatography was carried out using silica gel (Merck, particle size 40–63 microns). Melting points were measured on a Wagner & Munz PolyTherm A and are uncorrected. Infrared spectra were recorded on a THERMONICOLET Avatar 360 instrument using ATR. NMR spectra were recorded on a Bruker DRX500P (500.13 MHz ^1^H, 125.77 MHz ^13^C) or else on an Avance-III-600 (600.16 MHz ^1^H, 150.92 MHz ^13^C) spectrometer. Chemical shifts (δ) are quoted in parts per million (ppm) downfield of tetramethylsilane, using residual proton-containing solvent as internal standard (CDCl_3_ at 7.27). Abbreviations used in the description of resonances are: s (singlet), d (doublet), t (triplet), q quartet), br (broad). Coupling constants (*J*) are quoted to the nearest 0.1 Hz. Mass spectra were recorded with an Agilent 5973N detector coupled with an Agilent 6890N GC (GC–MS, 70 eV). HRMS spectra were recorded on a Finnigan MAT 95 (EI, 70 eV). Optical rotations were measured on a Perkin Elmer 341 LC polarimeter. Elemental analysis was performed on a Hekatech EA 3000.

**Thionocarbonate 9:** Diol **7** (10.0 mg, 39 μmol) and pyridine (6.2 mg, 78 μmol) were dissolved in dichloromethane (0.5 mL). The solution was cooled to 0 °C, and phenyl chlorothionoformate (10.3 mg, 60 μmol) was added. The solution was warmed to room temperature and stirred for 72 h. Then silica gel was added, and the solvents were removed in vacuo. Purification by flash chromatography (isohexane/ethyl acetate 2:1) afforded thionocarbonate **9** (4.9 mg, 17 μmol, 42%) as a colorless solid.

**9:** [α]_D_^20^ = −58 (*c* 0.50, CHCl_3_); Mp = 203 °C; ^1^H NMR (600 MHz, CDCl_3_) δ 4.62 (dd, *J* = 11.7, 3.8 Hz, 1H), 3.20 (s, 1H), 2.82 (dd, *J* = 15.8, 11.7 Hz, 1H), 2.63 (dd, *J* = 15.6, 3.6 Hz, 1H), 2.54 (dt, *J* = 12.1, 8.8 Hz, 1H), 2.48–2.43 (m, 1H), 2.19 (dd, *J* = 12.4, 7.2 Hz, 1H), 2.04 (s, 3H), 2.03–1.88 (m, 2H), 1.65–1.58 (m, 1H), 1.46 (s, 3H), 1.43–1.37 (m, 1H), 0.93 (d, *J* = 7.2 Hz, 3H) ppm; ^13^C NMR (150 MHz, CDCl_3_) δ 205.9 (C), 190.4 (C), 91.6 (C), 85.5 (CH), 64.3 (C), 58.4 (CH), 44.5 (CH), 41.1 (CH), 35.8 (CH), 31.2 (CH_2_), 27.4 (CH_2_), 24.9 (CH_2_), 23.0 (CH_3_), 19.4 (CH_3_), 16.9 (CH_3_) ppm; IR (ATR) ν: 2962, 2869, 1789, 1701, 1291, 1253, 1152, 977 cm^−1^; GC–MS: *m*/*z* = 296 [M]^+^; anal. calcd for C_15_H_20_O_4_S: C 60.79, H 6.80; found: C 60.88, H 6.92.

**Alcohol 3:** Epoxide **4** (100.0 mg, 454 μmol) and cobalt(II) acetylacetonate (23.3 mg, 91 μmol) were dissolved in THF (5 mL), and the solution was cooled to 0 °C. Oxygen was bubbled through the solution for 1 h, and phenylsilane (196.5 mg, 1.816 mmol) was added afterwards. After a reaction time of 22 h, ethyl acetate was added, and the mixture was filtered through silica gel. The filtrate was concentrated under reduced pressure, and the residue was purified by flash chromatography (isohexane/ethyl acetate 2:1) to yield alcohol **3** (63.2 mg, 265 μmol, 58%) as a colorless solid.

**3**: [α]_D_^20^ = +3 (*c* 0.54, CHCl_3_); Mp = 84 °C; ^1^H NMR (600 MHz, CDCl_3_) δ 3.23 (d, *J* = 7.2 Hz, 1H), 2.55 (ddd, *J* = 14.8, 7.4, 1.5 Hz, 1H), 2.40–2.32 (m, 1H), 2.03 (s, 3H), 1.96–1.89 (m, 1H), 1.85–1.80 (m, 2H), 1.76–1.68 (m, 2H), 1.64–1.51 (m, 2H), 1.38–1.31 (m, 1H), 1.24–1.19 (m, 1H), 1.13 (s, 3H), 1.11 (d, *J* = 7.2 Hz, 3H) ppm; ^13^C NMR (150 MHz, CDCl_3_) δ 208.2 (C), 74.8 (C), 64.0 (C), 60.0 (CH), 52.1 (CH), 44.8 (CH), 40.8 (CH_2_), 35.9 (CH), 34.6 (CH_2_), 25.4 (CH_2_), 24.4 (CH_2_), 23.3 (CH_3_), 19.9 (CH_3_), 15.5 (CH_3_) ppm; IR (ATR) ν: 3446, 2957, 2930, 2874, 1708, 1130, 852 cm^−1^; GC–MS: *m*/*z =* 238 [M]^+^; HRMS (EI) *m*/*z*: [M]^+^ calcd for C_14_H_22_O_3_, 238.1569; found, 238.1564.

**Allyl ether 11:** Methyltriphenylphosphonium bromide (131.5 mg, 368 μmol) was suspended in THF (1.5 mL), and a solution of sodium hexamethyldisilazide in THF (2.0 M, 172 μL, 344 μmol) was added. The mixture was stirred for 5 min at room temperature, and then a solution of alcohol **3** (29.0 mg, 122 μmol) in THF (1.5 mL) was added dropwise. After stirring for 5 min at room temperature, ethyl acetate was added, and the mixture was filtered through a plug of silica gel. Purification of the residue by flash chromatography (isohexane/ethyl acetate 2:1) afforded a mixture of olefin **10** and the cyclized product **11** as a colorless oil.

The mixture above and ytterbium(III) triflate (1.5 mg, 2.4 μmol) were dissolved in dichloromethane (1.5 mL) and stirred for 1 h at room temperature. Afterwards the reaction mixture was filtered through silica gel, and the filtrate was evaporated in vacuo. Flash chromatography (isohexane/ethyl acetate 10:1) of the residue afforded the oxygen-bridged bicyclic hydroazulene **11** (22.9 mg, 97 μmol, 80% over 2 steps) as a colorless oil.

**11:** [α]_D_^20^ = –36 (*c* 0.61, MeOH); ^1^H NMR (500 MHz, CDCl_3_) δ 5.02 (t, *J* = 1.6 Hz, 1H), 4.80 (s, 1H), 3.38 (d, *J* = 10.4 Hz, 1H), 2.30–1.38 (m, 1H), 2.20–2.07 (m, 1H), 2.06–1.92 (m, 1H), 1.86 (s, 3H), 1.82–1.72 (m, 2H), 1.71–1.60 (m, 2H), 1.54–1.45 (m, 1H), 1.41–1.36 (m, 1H), 1.28 (s, 3H), 1.22–1.13 (m, 1H), 1.10–1.00 (m, 1H), 0.89 (d, *J* = 7.3 Hz, 3H) ppm; ^13^C NMR (125 MHz, CDCl_3_) δ 147.2 (C), 113.2 (CH_2_), 88.0 (C), 82.9 (C), 69.8 (CH), 48.9 (CH), 46.7 (CH), 31.4 (CH_2_), 31.4 (CH_2_), 31.1 (CH), 30.7 (CH_2_), 26.0 (CH_2_), 24.3 (CH_3_), 19.3 (CH_3_), 17.1 (CH_3_) ppm; IR (ATR) ν: 3467, 3082, 1073, 2952, 2869, 890 cm^−1^; GC–MS: *m*/*z =* 236 [M]^+^. HRMS (EI) *m*/*z*: [M]^+^ calcd for C_15_H_24_O_2_, 236.1776; found, 236.1766.

**(−)-Oxyphyllol (1):** Olefin **11** (22.9 mg, 97 μmol) and palladium on charcoal (10 wt %, 5.1 mg) were placed in a flask, and methanol (2 mL) was added. The resulting mixture was stirred under hydrogen (1 atm) for 4 h at room temperature. Then the mixture was filtered through silica gel, and the solvents were removed in vacuo. Flash chromatography of the residue (isohexane/ethyl acetate 10:1) afforded **1** as a colorless oil (22.7 mg, 95 μmol, 98%).

**1**: [α]_D_^20^ = −51 (*c* 0.82, CHCl_3_); (Lit. [3]: [α]_D_^26^ = −40.25 (*c* 0.225, CHCl_3_)); ^1^H NMR (500 MHz, CDCl_3_) δ 3.69 (d, *J* = 10.1 Hz, 1H), 2.37–2.21 (m, 1H), 2.05–1.87 (m, 2H), 1.85–1.76 (m, 1H), 1.75–1.54 (m, 4H), 1.44–1.32 (m, 2H), 1.23 (s, 3H), 1.21–1.13 (m, 1H), 1.05–0.99 (m, 1H), 1.03 (d, *J* = 6.9 Hz, 3H), 1.02 (d, *J =* 6.6 Hz, 3H), 0.88 (d, *J* = 7.3 Hz, 3H) ppm; ^13^C NMR (125 MHz, CDCl_3_) δ 86.4 (C), 83.0 (C), 71.8 (CH), 49.3 (CH), 48.6 (CH), 32.4 (CH), 31.7 (CH_2_), 31.5 (CH_2_), 30.5 (CH), 29.2 (CH_2_), 25.9 (CH_2_), 24.3 (CH_3_), 18.4 (CH_3_), 17.5 (CH_2_), 17.1 (CH_3_) ppm; IR (ATR) ν: 3408, 2953, 2870, 1473, 1378, 1064, 1004 cm^−1^; GC–MS: *m*/*z =* 238 [M]^+^.

## Supporting Information

The Supporting Information contains ^1^H and ^13^C NMR spectra for compounds **1**, **3**, **9** and **11**.

File 1 ^1^H NMR and ^13^C NMR spectra.
