# Supplementary material for: A concise enantioselective synthesis of the guaiane sesquiterpene (−)-oxyphyllol
Source: Beilstein J Org Chem. 2013 Oct 8;9:2028–32. doi: 10.3762/bjoc.9.239 (PMC3817518; doi:10.3762/bjoc.9.239)
Supplement: File 1 — 1H NMR and 13C NMR spectra. [file Beilstein_J_Org_Chem-09-2028-s001.pdf]

Supporting Information

for

A concise enantioselective synthesis of the guaiane sesquiterpene (–)-oxyphyllol

Martin Zahel and Peter Metz*

Address: Fachrichtung Chemie und Lebensmittelchemie, Organische Chemie I, Technische Universität Dresden, Bergstrasse 66, 01069 Dresden, Germany

Email: Peter Metz - peter.metz@chemie.tu-dresden.de

* Corresponding author

^{1}H NMR and ^{13}C NMR spectra

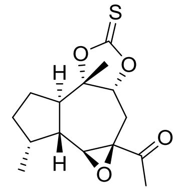
S
O
O
H
H
O
O

9

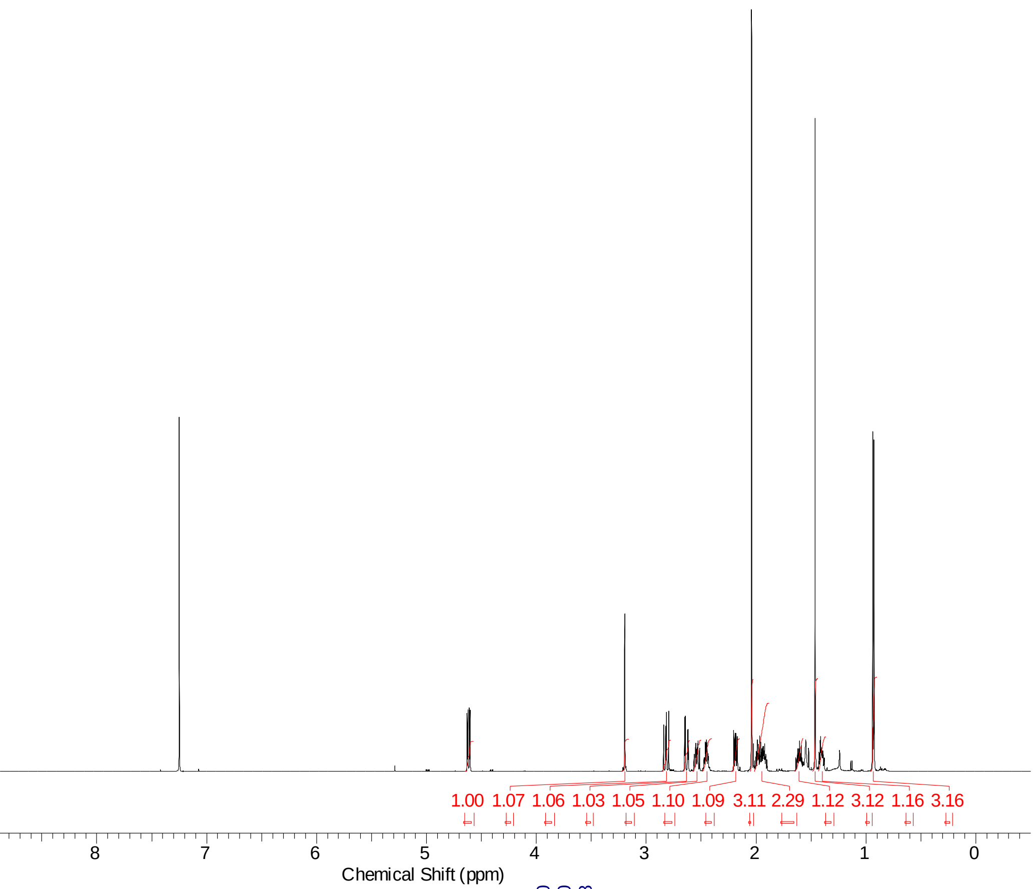
1.00 1.07 1.06 1.03 1.05 1.10 1.09 3.11 2.29 1.12 3.12 1.16 3.16
10
9
8
7
6
5
4
3
2
1
0
Chemical Shift (ppm)

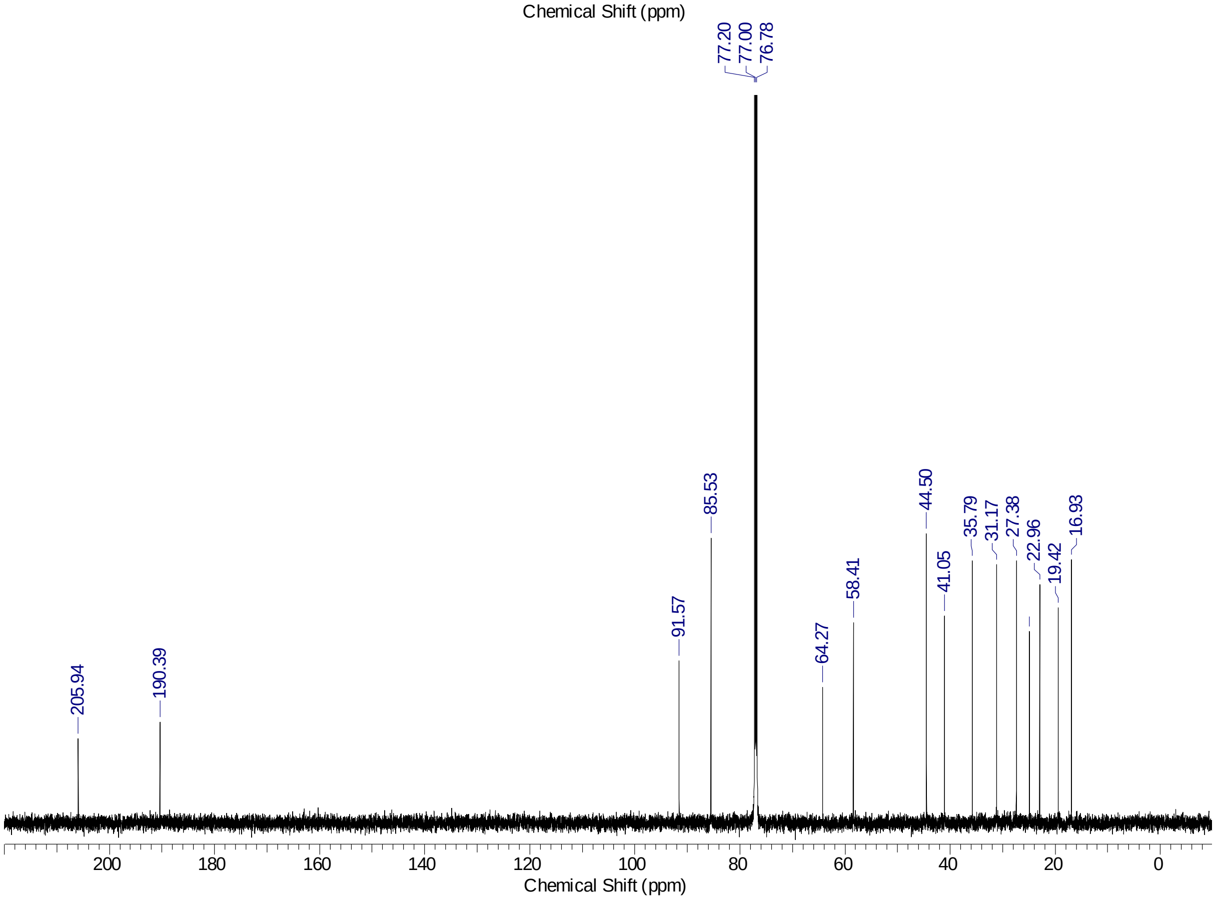
77.20
77.00
76.78
205.94
190.39
91.57
85.53
64.27
58.41
44.50
41.05
35.79
31.17
27.38
22.96
19.42
16.93
200
180
160
140
120
100
80
60
40
20
0
Chemical Shift (ppm)

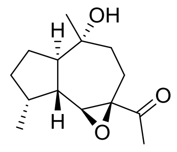
OH
H
H
O
O

3

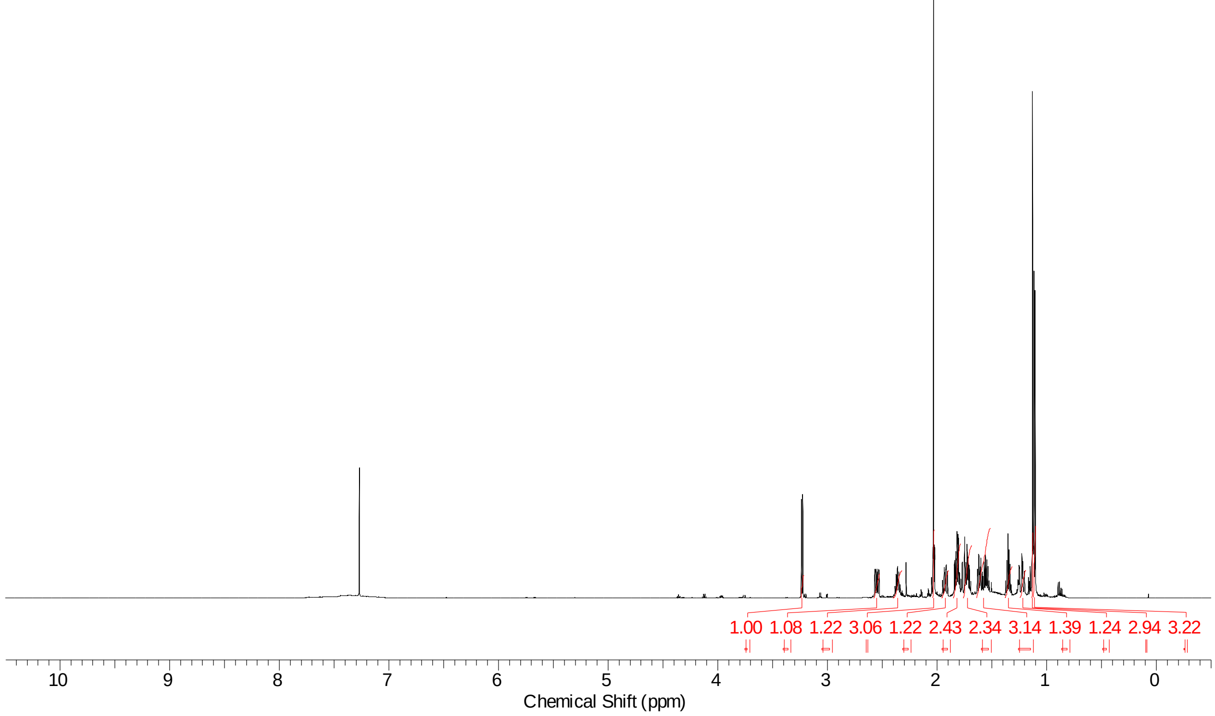
1.00 1.08 1.22 3.06 1.22 2.43 2.34 3.14 1.39 1.24 2.94 3.22
10 9 8 7 6 5 4 3 2 1 0
Chemical Shift (ppm)

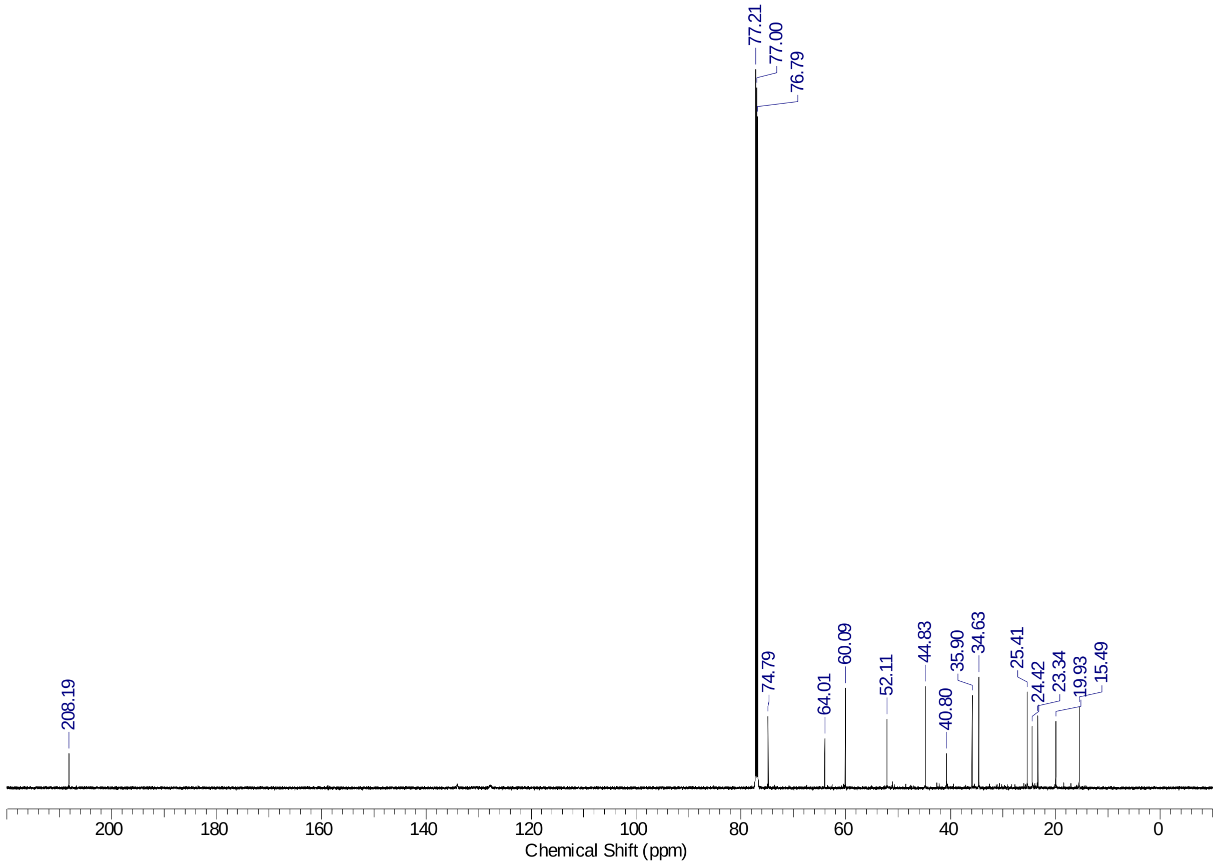
77.21
77.00
76.79
208.19
74.79
64.01
60.09
52.11
44.83
40.80
35.90
34.63
25.41
24.42
23.34
19.93
15.49
200 180 160 140 120 100 80 60 40 20 0
Chemical Shift (ppm)

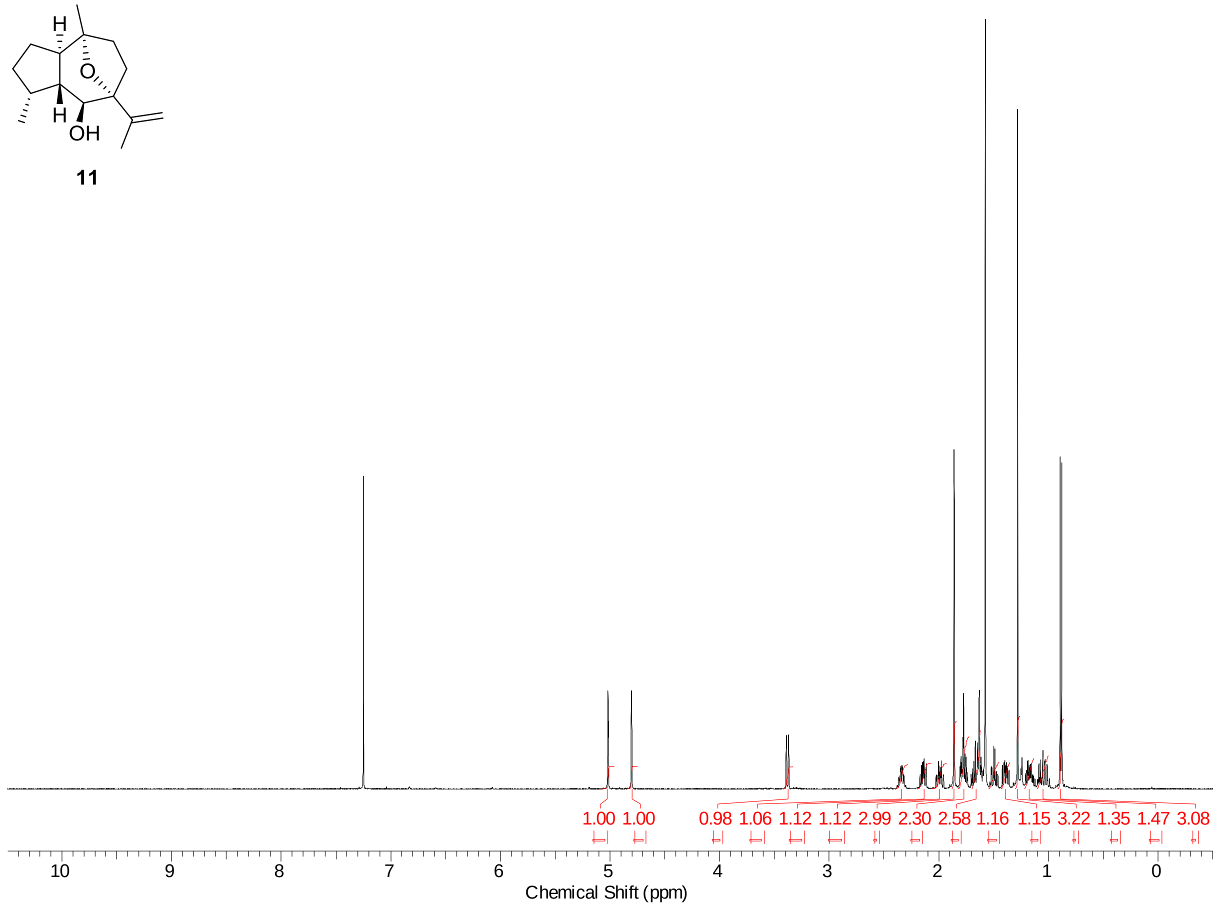
H
O
H
OH
11
1.00 1.00 0.98 1.06 1.12 1.12 2.99 2.30 2.58 1.16 1.15 3.22 1.35 1.47 3.08
10 9 8 7 6 5 4 3 2 1 0
Chemical Shift (ppm)

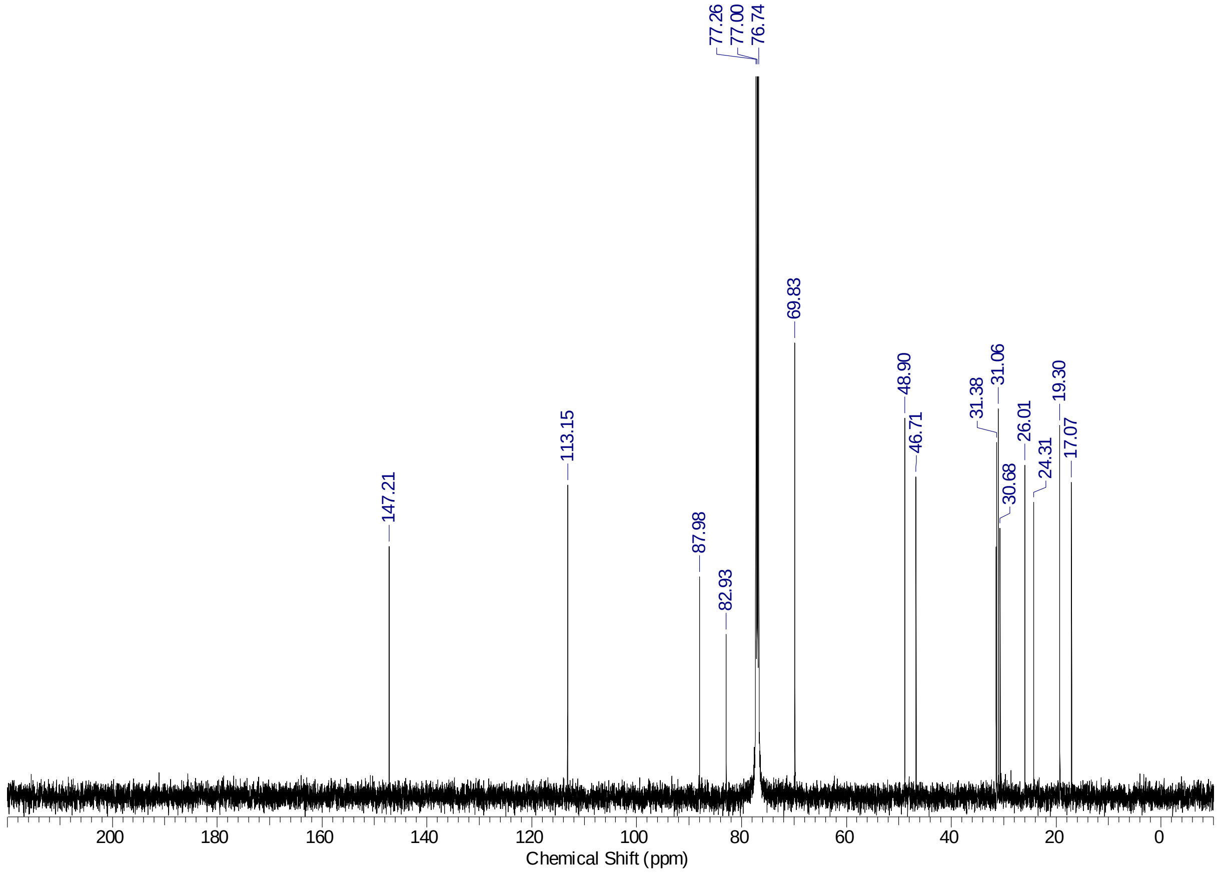
77.26
77.00
76.74
69.83
48.90
46.71
31.38
31.06
30.68
26.01
24.31
19.30
17.07
147.21
113.15
87.98
82.93
200 180 160 140 120 100 80 60 40 20 0
Chemical Shift (ppm)

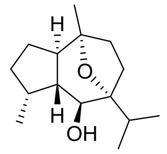
H
O
H
OH

1

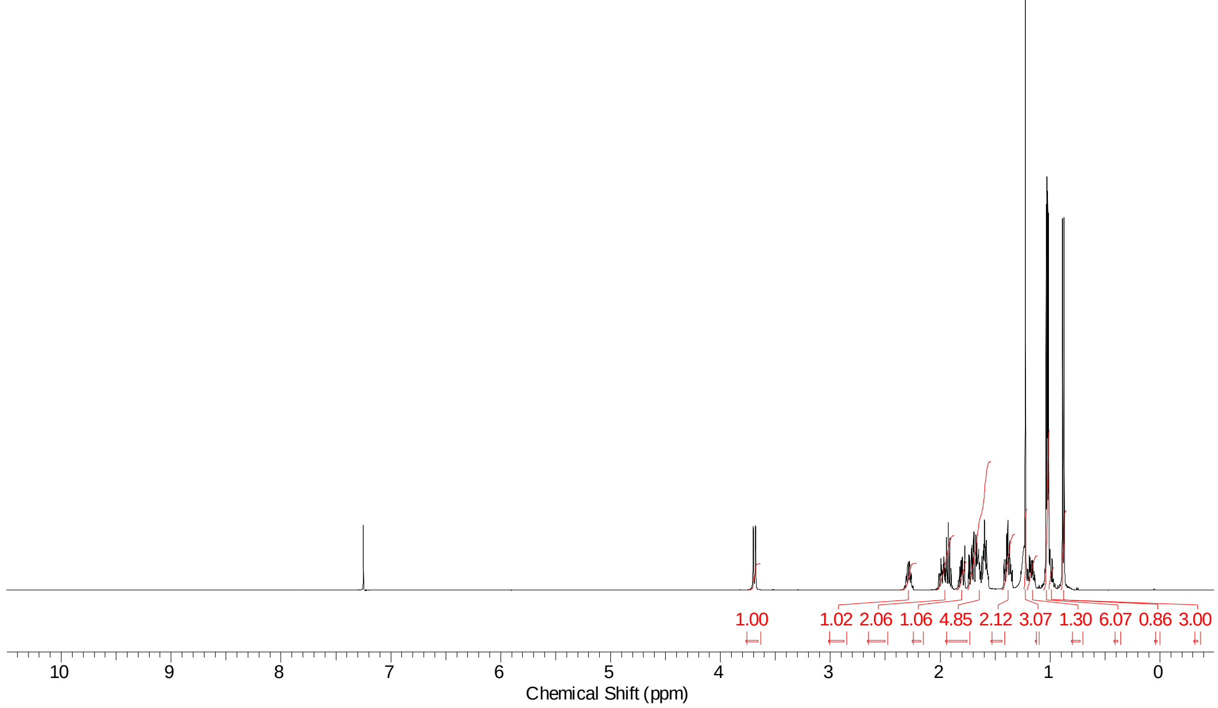
1.00
1.02
2.06
1.06
4.85
2.12
3.07
1.30
6.07
0.86
3.00
10
9
8
7
6
5
4
3
2
1
0
Chemical Shift (ppm)

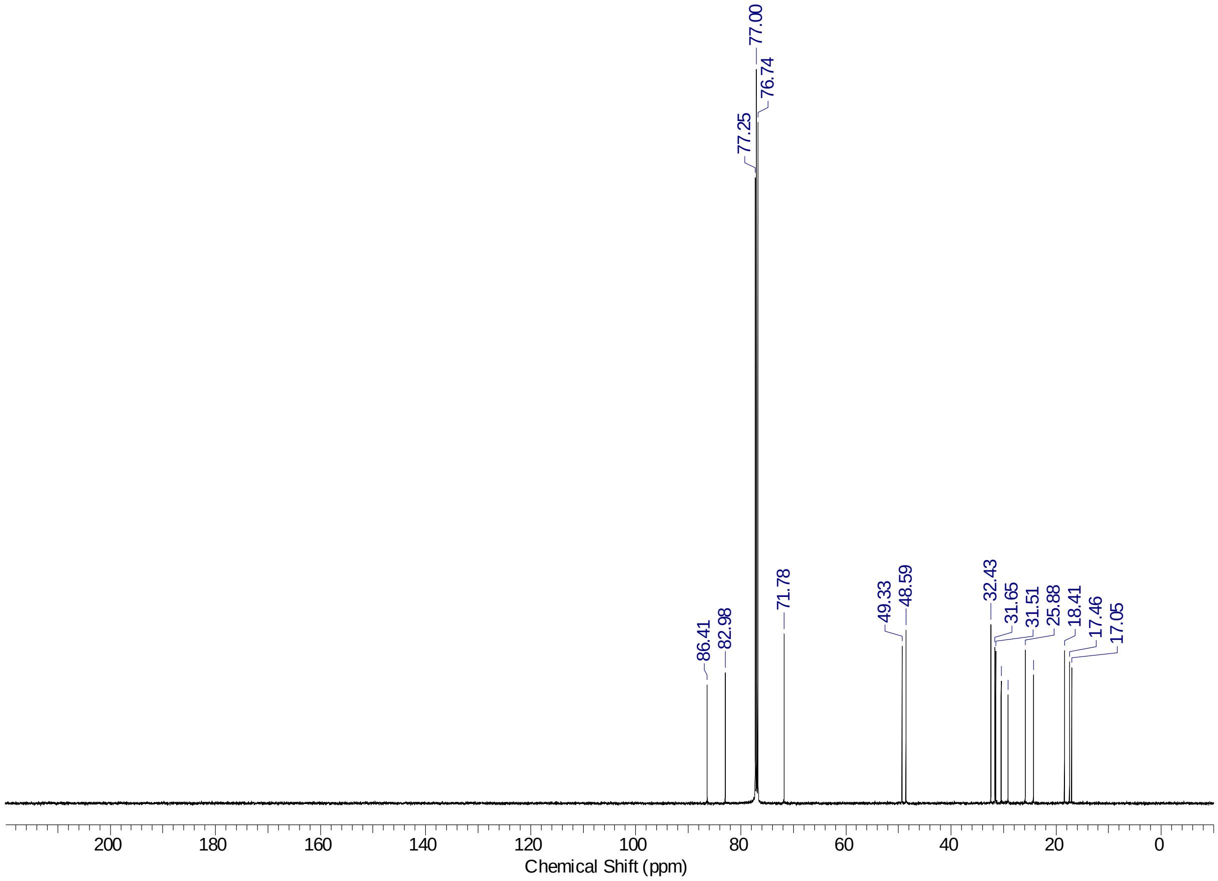
77.00
76.74
77.25
86.41
82.98
71.78
49.33
48.59
32.43
31.65
31.51
25.88
18.41
17.46
17.05
200
180
160
140
120
100
80
60
40
20
0
Chemical Shift (ppm)
